# Pulmonary Consumption; Its Prevention and Cure Established on New Views of the Pathology of the Disease

**Published:** 1842-07-01

**Authors:** 


### Pulmonary Consumption: its Prevention and Cure established on new Views of the Pathology of the Disease. By *Henry Gilbert*, Member of the Royal College of Surgeons, London. London: Henry Renshaw, 1842.

Mr. Gilbert conceives that if the attention of the public can be drawn to the subject of consumption, and impressed with the necessity and value of preventive measures, his work will not have been written in vain. We are of the same opinion, and shall give a brief account of the contents of the present volume.

### Statistics of Consumption.

The first chapter is devoted to statistical observations on the disease. Some of the facts are striking. The mortality from phthisis has been estimated at from a *fourth* to a *fifth* of the entire mortality of the kingdom. The following are the facts deducible from the first annual Report of the Registrar General.

The total number of deaths registered in England and Wales, from July 1st to December 31st, 1837, both inclusive, amounted to 148,701. Of this number, 27,754 were the result of consumption of the lungs, of whom 12,968 were males, and 14,786 were females. We therefore find that, according to this authentic report, consumption caused twenty per cent. of the total number of deaths, thus confirming Dr. Abercrombie's opinion, that one-fifth part of all deaths are the consequence of this fearful malady. But if we take away 12,691 deaths from old age, and 4845, which were violent, in all 17,536, we shall then find that consumption produced upwards of a fifth part of all those which resulted from disease, thus bearing out the opinion of Dr. Young and Dr. Woolcombe, that one-fourth part of the deaths occurring from disease is the result of phthisis. The whole evidence is therefore singularly unanimous.

According to the Report of the Registrar-General, pulmonary consumption destroyed more human lives during the six months referred to, than did cholera, influenza, small-pox, measles, ague, typhus-fever, hydrophobia, apoplexy, hernia, colic, diseases of the liver, stone, rheumatism, ulcers, fistula, and mortification! Those diseases proved fatal to 26,881 persons, consumption to 27,754.

*Influence of Localities.*—Mr. Gilbert first speaks of the Metropolis. The total number of deaths that occurred in this vast city, from July 1st to December 31st, 1837, both inclusive, was 24,959, being nearly at the rate of one every ten minutes. Of this number 3877 were the consequence of consumption, namely 1947 males, and 1930 females, so that London loses about one soul every hour throughout the whole year by this malady. One circumstance must, however, be observed: we have already seen that phthisis produces twenty per cent of the total number of deaths occurring in England and Wales, or in other words, that every fifth death, from whatever cause, is the result of this malady. But from the statement relative to London individually, we perceive that here scarcely every sixth death is produced by consumption. Although large cities are proverbially unhealthy, and pulmonary affections generally considered to prove very fatal in such localities, nevertheless, there are few towns or rural districts so free from phthisis, in proportion to their population, as London.

Mr. Gilbert thinks that phthisis runs its course more slowly in London than in many of the most healthy rural districts. He compares with London Devonshire.

From July 1st to December 31st, 1837, both inclusive, there were 4801 deaths in Devonshire. Out of this number 835 were the consequence of phthisis; namely, 389 males and 446 females. Thus we find, that of the deaths occurring in this favourite retreat for the consumptive invalid, one-fifth were the result of consumption, whereas in the smoky metropolis there were not quite one-sixth. Mr. Gilbert thinks that if patients go to Devonshire *to die* of consumption, they come to London in still greater proportion.

From Devonshire he passes to Birmingham and Leeds.

From July 1st to December 31st, 1837, both inclusive, the total number of deaths that occurred in Birmingham was 1459, of which 354 were the result of phthisis, namely, 190 males and 164 females. The number of consumptive deaths in Birmingham, as compared with those in London, appears not a little surprising. In the metropolis less than one-sixth of the aggregate mortality is referable to this malady, whereas in Birmingham every fourth death is attributable to its ravages.

During the period above cited, namely, from July 1st to December 31st, 1837, both inclusive, the total number of deaths which took place in Leeds amounted to 1582, out of which 335 were the results of phthisis, namely, 161 males and 174 females. We, therefore, here find the mortality from consumption comparatively great, upwards of a fourth part of the whole deaths resulting from it.

In Norfolk and Suffolk the total number of deaths occurring from July 1st to December 31st, 1837, both inclusive, amounted to 6017. Of these 1306 were the consequence of consumption, namely, 582 males and 724 females. A fourth part of the population would therefore appear ultimately to fall the victims of this malady in this district; so that pulmonary phthisis may be fairly considered more prevalent in Norfolk and Suffolk than in Devonshire or London.

*Increase or Decrease of Consumption.*—In a work on statistics by Mr. Marshall there is shewn the annual mortality in the metropolis during a period of two hundred and four years, namely, from 1629 to 1831, both inclusive. Mr. Gilbert observes:—"Availing ourselves of this work, we shall go back to the year 1629, and calculate the proportional share of deaths produced by consumption, from that period, during intervals averaging ten years, up to 1830:

| YEAR. | TOTAL NUMBER OF DEATHS. | DEATHS FROM CONSUMPTION. | PROPORTION FROM CONSUMPTION. |
|---|---|---|---|
| 1629 | 10,554 | 1,827 | One-fifth. |
| 1647 | 14,059 | 2,423 | One-fourth. |
| 1657 | 15,046 | 2,757 | One-fifth. |
| 1667 | 15,842 | 3,087 | One-fifth. |
| 1677 | 19,067 | 3,272 | One-fifth. |
| 1687 | 21,460 | 3,473 | One-sixth. |
| 1701 | 20,471 | 2,678 | One-seventh. |
| 1710 | 24,620 | 2,706 | One-ninth. |
| 1720 | 25,454 | 3,054 | One-eighth. |
| 1730 | 26,761 | 3,728 | One-seventh. |
| 1740 | 30,811 | 4,919 | One-sixth. |
| 1750 | 23,727 | 4,543 | One-fifth. |
| 1760 | 19,830 | 3,776 | One-fifth. |
| 1770 | 22,434 | 4,594 | One-fourth. |
| 1780 | 20,517 | 4,889 | One-fourth. |
| 1790 | 18,038 | 4,852 | One-third. |
| 1800 | 23,068 | 5,721 | One-fourth. |
| 1810 | 19,893 | 5,427 | One-third. |
| 1820 | 19,348 | 3,959 | One-fourth. |
| 1830 | 21,645 | 4,704 | One-fourth. |

From the above table we may infer, that pulmonary consumption has of late years decreased in the metropolis; for, from the year 1770 up to 1830, it averaged one-fourth, and sometimes as high as one-third of the whole mortality, whereas now and for some years back, it has not exceeded one sixth. The variations occurring during the period embraced in the table, are, however, very considerable, and shew that in 1701, 1710, 1720, and 1730, the deaths from phthisis were proportionally fewer than they even are now. Sir James Clark, in his Treatise on Consumption, gives a table, constructed from data contained in Mr. Marshall's work on the Mortality of the Metropolis, and remarks that 'from 1700 to 1750 the deaths from consumption increased from four to six in every thousand of the population,' and that since the last period they have remained stationary. But in coming to the conclusion that this disease has increased, he must surely be aware that he has selected, as a period of comparison with subsequent dates, one when the number of consumptive cases was singularly low. Had he gone back for a few years, he would have found the number of phthisical cases averaging one-fifth of the whole, and in 1687 they were, as at the present time, one sixth."

Mr. Gilbert thinks, however, that, during the last ten years, consumption has increased in the upper classes, but decreased in the lower. Sir James Clark is of the same opinion with reference to the upper classes.

*Influence of Climate.*—Consumption is not rife in cold countries. It is rare in Russia and in Canada. Mr. Marshall gives a table, shewing the mortality in each of the twenty-four provinces of this country, and in the city of Stockholm, in the year 1820, in which table he exhibits the various diseases from which the mortality ensued. From this account it appears, that during the year referred to there were 31,572 male deaths, of which number, only 3110 were the result of consumption; being only one-tenth of the whole. Out of the female population there occurred 31,358 deaths, of which 3195 were from phthisis; constituting one-ninth part of the whole. Now as one-fifth part of the population die in England, where the cold is not nearly so intense, it follows that mere cold, if uniform and steady, is not conducive to consumption. But in a variable climate the cold months are the most fatal months. Dr. Heberden has furnished some Tables which conclusively establish this.

He gives a statement of the mortality from consumption, in London, during a period of ten years, namely, from January, 1763, to December, 1767, and from January, 1795, to December, 1799, these years consequently inclusive. The facts were extracted by him from the weekly bills of mortality, and shew the variations every week, during the whole period. The following is a statement of the whole number of deaths from phthisis during the ten years, shewing the months when they took place:—

| | | | |
|---|---|---|---|
| January | 4363 | July | 3249 |
| February | 4527 | August | 2825 |
| March | 4634 | September | 2994 |
| April | 4227 | October | 3521 |
| May | 4043 | November | 3711 |
| June | 3604 | December | 4516 |

We therefore find the occurrence of death from consumption more frequent during the Winter than Summer months. The variations, during the four seasons of the year, are as follow:—

| | |
|---|---|
| Winter | 13,406 |
| Spring | 12,904 |
| Autumn | 10,226 |
| Summer | 9,678 |

In countries where the Winter is not so severe as in Great Britain, the Spring is generally most fatal to the consumptive patient. This has been illustrated by M. Benoiston, as follows. Out of 12,668 deaths from phthisis, occurring at Milan, Paris, and in the adjacent country, the proportion in the different seasons was as follows:—

| | | |
|---|---|---|
| Autumn | 3001 | 12,668 |
| Winter | 3109 | |
| Spring | 3482 | |
| Summer | 3076 | |

*Heat*—Mr. Gilbert is of opinion does not particularly induce consumption. If it did, why should consumption be comparatively rare in the East Indies?

*Humidity.*—This too he contends does not favour the production of phthisis, but rather the contrary. Among other arguments, he cites two Tables from Dr. Lombard of Geneva, the inference from which is, that workmen surrounded by a hot dry atmosphere yield more readily than other workmen, in the proportion of 127 to 114; from which it may be inferred, that if a moist atmosphere is a preservative against consumption, so hot dry air may be considered as a cause of the disease.

*Influence of Occupations.*—It is well known that the inhalation of metallic or other particles, disseminated in the air, disposes to consumption. Perhaps the two following are the most striking examples of the fact. M. Benoiston de Chateauneuf informs us that the whole of France, and the greater part of the continent of Europe, is supplied with gun-flints from a single parish in France, that of Meusnes, containing a population of twelve hundred souls, of whom, about three hundred families are occupied with the flint manufacture. The French government alone consumes ten millions annually, and the exports amount to two hundred and eighty millions. This singular trade, however, has not been acquired without a terrible sacrifice of life and health on the part of the workmen. About the beginning of the last century, before flints were used for muskets, the mortality of the parish was one in $33\frac{1}{4}$; of the births, one-half survived till the eighteenth year; and the mean duration of life was $24\frac{1}{4}$ years. But after the establishment of the manufacture of flints, the mortality became one in $22\frac{3}{4}$; half of the births were cut off by the fifth year, and the mean term of life was reduced to $19\frac{1}{6}$ years. The cause of this enormous increase in mortality, according to the author, was consumption, arising from the inhalation of flint-dust.

Dr. Knight says, that the grinders of Sheffield "altogether amount to about two thousand five hundred; of this number, one hundred and fifty, namely, eighty men and seventy boys, are fork-grinders; these grind dry, and die from twenty-eight to thirty-two years of age. The razor-grinders grind both wet and dry, and they die from forty to forty-five years of age. The table-knife-grinders work on wet stones, and they live to betwixt forty and fifty years of age." On comparing the diseases of the grinders with those of other mechanics, in Sheffield, he found, that two hundred and fifty-four grinders laboured under disease of the chest; while only fifty-six were similarly affected in the same number of workmen among other trades.

Butchers, knackers, tanners, and tallow-chandlers are remarkably exempt from phthisis.

*Sex.*—Mr. Gilbert has constructed a table from the Registrar-General's Report, with a view of determining the influence of sex. It appears that, from July 1, to December 31, 1837, making our observations on England and Wales as a whole, consumption was more fatal among females than males. The total number of female deaths was less by 1,617 than that of males, but the number of female consumptive cases exceeded that among males by 1,818. But, to be more particular, in London, the mortality seems equally divided between the sexes; in Devonshire, a greater proportion of females fall victims to this disease than males; and, again, in Birmingham the proportions vary but little. M. Benoiston de Chateauneuf infers, from his statistical observations, that phthisis is, in France, much more common among females than males. Out of 1,554 phthisical deaths registered in four of the Parisian hospitals, (the Hôtel Dieu, La Pitié, La Charité, and Cochin,) 745 were men, and 809 women; while, of the admissions, 26,055 were men, and 16,955 women; so that the proportional deaths from phthisis relatively to the admissions are

28·5 per thousand for men, but 47·5 per thousand for women. But, observations of this sort are obviously defective.

*Age.*—Mr. Gilbert thinks consumption more prevalent in infancy than is imagined. It is overlooked in consequence of their swallowing their expectoration, or being supposed to labour under hooping-cough. M. Guersent, an experienced physician, attached to the Hôpital des Enfans Malades, in Paris, a charity admitting no patient under the first or above the sixteenth year, gives it as his opinion, that tubercles existed in two-thirds, or even five-sixths of the bodies which he examined.

M. Bayle has drawn up a table to shew the different ages at which death occurred among a hundred phthisical patients who died in the Hospital of La Charité, at Paris. From this it appears that, out of one hundred deaths from consumption, thirty-three only were below the age of thirty and sixty-seven above it, of which last number forty-four were above the age of forty.

Dr. Alison, of Edinburgh, says, that "in the practice of the New Town Dispensary here, there have been fifty-five deaths from phthisis in the last two years. Of these, eight occurred before fifteen years of age, thirteen between fifteen and thirty, thirty-four after thirty, and of these last twenty-four after forty."

Age then is no protection.

### PATHOLOGY OF CONSUMPTION.

After giving a brief account of the healthy process of nutrition, and of the manner in which organizable matter is absorbed, through the lacteals into the blood, Mr. Gilbert proceeds to observe, that a large proportion of our food is either inorganizable, or not changed by the digestive organs so as to render it fit for assimilation. Now sometimes, he says, a portion of this inorganizable matter is taken up by the lacteals and circulated in that state.

These grains of inorganizable matter, so circulating, seem to produce no local mischief, often, however, rendering the whole system irritable. But when they are deposited in any organ, they there produce effects exactly similar to what would result from the deposition of gun-shot. It is this inorganizable matter, absorbed from the alimentary canal, and circulated with the blood, that constitutes the seeds of pulmonary consumption. It is the irritation produced by these seeds when deposited in the lungs, that leads to all the melancholy consequences attendant on this dreadful malady.

Pulmonary consumption, or tubercular phthisis, cannot take place till these seeds are absorbed, and circulate with the blood.

In medicine, these seeds are generally known by the name of tubercular matter, and when deposited in the lungs in separate masses, they are termed *tubercles*, and the resulting disease is denominated consumption of the lungs.

It may, however, be here observed, that when tubercular or inorganizable matter becomes deposited in any of the lymphatic glands, or in the joints, the disease is termed scrofula. If, on the other hand, it is arrested in its passage through the mesenteric glands, already referred to, then the name of tabes mesenterica is employed to distinguish it. It must, therefore, appear obvious, that these three diseases are all radically one and the same, depending entirely on the presence of inorganizable matter, and differing only in locality.

Mr. Gilbert anticipates a natural question—what proof can be adduced of this inorganizable matter coming from the intestine? His answer will be found in the following passage, which it is only fair to give entire.

"In the first place, every one will allow that a great portion of the contents of the alimentary canal consists of inorganizable matter. This is indisputable. Secondly, the lacteals, which in the healthy state only take up chyle, may, when in a morbid condition, also absorb inorganizable matter. Numberless experiments have been instituted on the subject of absorption, and many with the view of detecting whether or not the lacteals admit any substance except chyle. Lister and Musgrave shewed that the lacteals do absorb inorganizable matter; and Haller, Hunter, and Cruickshank, have all given similar evidence. We cannot therefore deny, that they, when in a morbid state, may absorb a portion of that inorganizable matter, and, in fact, the experiments instituted by the above-named scientific authorities prove that they do. Dr. Ayre tells us, plainly and distinctly, that 'diseased mesenteric glands occur in children from the acrid condition of the duodenal contents.' Now the disease found in the mesenteric glands of children is neither more nor less than a collection of inorganizable matter, which we may fairly presume, from Dr. Ayre's statement, came from the duodenum or alimentary canal. We therefore see that inorganizable matter may be absorbed by the lacteals, and I can have no difficulty whatever in shewing its frequent existence in these vessels, with, I think, demonstrative proof that it came from the prima via or alimentary canal. Dr. Carswell, in his note explanatory of figure i., plate 3, representing tubercular disease, shews 'the lacteals dilated, and filled with tuberculous matter, passing from the intestine into, and out of, the mesenteric glands, many of which are enlarged. All of them contain a greater or less quantity of tuberculous matter; one of them is completely filled with it. The lacteals are represented, arising from the ulcerated follicular gland. One of the branches was injected with mercury, the progress of which was soon arrested by the tubercular matter accumulating in front of the metal.' The preceding are Dr. Carswell's own words. They prove a great quantity of inorganizable matter in the lacteals close to the intestine, and in such quantity, and in such a locality, that it is directly contrary to physiology to ascribe any other source to it than the intestine. It is not a little singular that the lacteals so filled with tubercular matter are described as 'arising from the ulcerated follicular gland,' a circumstance shewing that they were in a morbid condition, as I have represented them to be, when indiscriminate absorption is going on.

But the frequency of inorganizable matter in the lacteals and mesenteric glands, as compared with other parts, evidently points them out as its first channel, and through which it reaches the blood. Meckel, who is a very high authority in questions of pathological anatomy, tells us, that the mesenteric glands are the parts most subject to tubercular degeneration, more so even than those of the neck, groin, or axilla.

On the whole, it must be allowed that it may come from the alimentary canal by the lacteals, and that it does so is rendered very manifest, by the experiments already referred to. Moreover, there is no other way of accounting for its origin, and here is a plain, simple solution of the mystery, supported by physiology, anatomy, and every-day observation.

And as the blood receives its constituent parts from the lacteals, if we can, in the next place, find tubercular matter in that vital fluid, I think there can be no additional proof required of its original source. That inorganizable or tubercular matter does become mixed with the blood, cannot now be denied." 95.

We must confess that the proofs adduced are not quite satisfactory to our minds. That tubercles consist of inorganizable matter cannot be doubted—that *that* matter is deposited from the blood is very far from improbable, nay is most likely—that, the lungs being part of the great apparatus of assimilation is linked with the stomach and intestines, and prone to suffer when their office is imperfectly performed, must be admitted —that inorganizable matter *may* be taken up from the alimentary canal, and *may* be deposited as tubercle in the lungs or elsewhere must be admitted too—but we think it would be difficult to conceive that this is the sole source of tubercle. The blood is not alone composed of new materials; the lymph from all the tissues and organs is poured into it as well. In that cachectic state which precedes or attends phthisis, we may suppose that inorganizable matter may be derived from this quarter, and in fact that both the new materials and the old may furnish the pabulum of tubercles. In the present state of our knowledge this appears to us the most consistent hypothesis. The subject is one that merits, and, we dare say, will receive the attention of pathologists. Careful experiments and accurate analysis of the blood may do much towards solving the riddle.

Carrying out his view, Mr. Gilbert lays the mischief at the door of the lacteals. "What, then," he asks, "is their peculiar hereditary organization that induces the tubercular cachexia, and which ultimately leads to consumption in so great a proportion of cases? This, I consider, may depend on two circumstances. No organ can perform its office without the influence of nerves—therefore the lacteals must be dependent on their nerves for the due execution of their office; and as the nerves of the eye, ear, or any other organ, may be hereditarily malformed, the like may occur in those of the lacteals. Although we cannot always, in cases of partial deafness, when it results from some imperfections in the auditory nerves, detect the cause by investigating the structure of those nerves, in consequence of their exquisite delicacy, still we must grant that, in many instances, some organic peculiarity is the cause of the deficient sense. We cannot but admit that such may be the case with the nerves of the villi. But again: in the eye and ear the respective nerves are very often perfectly developed, and capable of performing their functions, yet, from some morbid condition of the surrounding parts, they cannot act. The optic nerve is in general perfectly sound in cataract, but, from the opacity of the crystalline lens, vision is nevertheless obstructed. The ear is often similarly circumstanced. Thickening of the tissue in which a nerve is deposited will render its action deficient, and I have no doubt but this is sometimes a cause of the deficiency of power in the nerves of the villi. This condition would not, therefore, depend on any diseased state of the nerves, but on the peculiar organization of the surrounding parts, whereby the nerves cannot exercise their function to the required extent.

Similar circumstances would, as respects the lacteals, by depriving their nerves of due power, also disqualify them from discriminating between organizable and inorganizable matter, and thus predispose the individual so circumstanced to pulmonary consumption. In endeavouring to prevent the disease, it is to the original cause that we must direct our attention with any reasonable prospect of success."

In non-hereditary phthisis, he continues, the lacteals may lose their discerning faculty, by exposure to the influence of unnatural agents; and when so exposed, the result will be precisely similar to what took place from hereditary disease—namely, the absorption of inorganizable matter. The changes that take place in their structure may be exactly the same as what we found as an hereditary development, although, in this instance, other circumstances may also lead to the absorption of the seeds of consumption.

Whatever will destroy the sense of discernment in the lacteals, will lead to the absorption of inorganizable matter. The causes of this may be divided into direct and indirect. The direct causes of this morbid action are, with one solitary exception, the continued or repeated influence of matter coming in contact with the villi, which is unnatural to them. These are,—

Improper diet,
Injudicious use of medicine,
Inordinate indulgence in spirituous liquors; and,
Excessive fasting.

The indirect causes of the absorption of inorganizable matter are, whatever circumstances may impair the process of digestion. And he cites as samples, deficient exercise, and excessive labour.

After some details to which we need not advert, Mr. Gilbert adds—

"From the foregoing views, we would therefore attribute the production of phthisis, in many cases, to functional derangement of the stomach, and other organs connected with digestion. This, as well as some other causes, induces irritation, inflammation and thickening, in the intestinal villi, by which they ultimately lose their delicacy of sense. Absorption of inorganizable matter is the consequence. The influence of powerful narcotics is another cause of phthisis, but one which, I believe, acts directly in subduing the sense of discernment peculiar to the lacteals, without producing any irritation on them. The ultimate effects are, however, exactly the same as in the preceding case.

I have paid considerable attention to the state of the lacteals in those who have fallen victims to phthisis, and in subjects with tuberculated lungs. In examining the villi, inflammation has often been most apparent, and their natural appearance in other respects altered. And I have reason to believe, that the inflammation and ulceration so frequently found in the mucous coat of the duodenum, jejunum, ilium, and colon, in consumptive patients, often commence in the lacteals, although there can exist no doubt but that tubercular deposit in this membrane often aggravates such a condition, and may, in fact, be the sole cause thereof." 125.

A *section* is occupied with the *circumstances which lead to the deposition of tubercles in the lungs.*

This he attributes to two distinct causes—increased vascular action in the pulmonary tissue, and mechanical obstruction. By the former, the smaller vessels are distended with red blood, and if this state is kept up, increased secretion of mucus, and eventually of pus, is the result. And if this take place in an individual having inorganizable matter mixed with his blood, this matter, the seeds of consumption, necessarily constitutes part of the contents of the smaller vessels, as also of the increased secretion or effusion. Mr. Gilbert does not doubt that it is often mechanically arrested in the capillaries, and then thrown into the air-cells with the secretion, there it frequently remains. This having taken place, the individual is now in the first stage of the malady. Tubercular matter may be often seen in the air-cells of those who have sunk under phthisis, mixed up with the secretions. It is a great object, then, in those predisposed to phthisis, to keep down vascular action in the lungs.

Mechanical obstruction plays another prominent part in the production of phthisis. And such may be produced by a compressed and contracted state of the chest, whether from the effects of posture, the pressure of corsets, malformation, or whatever other cause.

### DIAGNOSIS.

From amongst some sensible remarks on this head, we select a note containing M. Fournet's researches on the expectoration of phthisis. The value of the expectoration, remarks M. Fournet, as a means of diagnosing phthisis, has been variously represented by medical writers. If, however, we consult what has been written on the subject, we discover nothing but vagueness and uncertainty. The expectoration appears under a great many different forms, none of them of any value, because none of them fixed or constant. This variety in the characters of the expectoration is more especially remarkable, according as we approach the first stage of phthisis. The expectoration is sometimes wanting altogether, even in the most advanced periods of the disease. At other times it retains the mere salivary appearance throughout the entire course of the disease, with the exception of the catarrhal periods. It is sometimes a little viscid, sometimes clear, sometimes turbid, sometimes homogeneous, sometimes mixed with small greyish, blackish, or opaque, dull, white sputa, which are occasionally met in the expectoration of individuals, whom we have no reason whatever to consider phthisical. The sputa are sometimes full of blood; at other times, from the very commencement of the disease, they are whitish, thick, and as it were a little pearly; in some patients they put on the appearance of small masses, with uneven, jagged edges; whilst in others, those small masses are regularly rounded. Sometimes, when catarrh occurs, the expectoration assumes all the characters observed in the sputa of bronchitis. The expectoration may be wanting, even in cases where the lungs are the seat of large cavities. Amidst such a variety of forms and characters it is scarcely possible to meet any sufficiently fixed to merit attention on the part of the practitioner. In a considerable number of phthisical patients, however, the expectoration goes through the following stages:—At first, it is merely salivary, then a little viscid, but transparent and homogeneous; sometimes marked, at this period and a little later, with some streaks of blood, then becoming a little more abundant; towards the transition from the first to the second stage of phthisis, instead of the clear, frothy fluid hitherto observed, we observe some whitish, opaque points, about the size of a pin's head, of a rounded or flattened form, which give a pearly appearance to the ordinary expectoration. These points increase in number and size, resembling grains, of a dull, white colour, and sometimes of a dark grey, which, becoming every day more numerous, and larger, after a certain time put on the appearance of small, irregularly-rounded masses, jagged at their edges, and varying in size from that of a lentil to that of a one or two franc piece. These small opaque masses seem to float in the midst of a transparent, viscid fluid; they are sometimes a little streaked with blood; we are then coming towards the end of the second stage of phthisis: sometimes cavities are already formed in the lungs; then, to complete the series of its changes, the expectoration has only to take on the purulent character, which it is known to have at this period. M. Fournet positively asserts that the *physical characters* of the expectoration can furnish no assistance whatever in diagnosing the first stage of phthisis.

Our author also quotes from M. Fournet, his account of the modifications of the respiratory murmur in the first stage of phthisis. It may be new to many of our readers, and squares very closely with the fact.

1. *Inspiratory murmur.*—Increased intensity of this murmur, which increase is exactly proportioned to the physical alteration of the lung.

Diminished duration of this murmur, which takes place at the same time as the increased intensity of it.

The character of this murmur, with respect to softness, dryness, and humidity, also undergoes a change. In the first period of the disease the inspiratory murmur is dry, rough, and, as it were, of difficult production. If we auscult the anterior part of the chest from below upwards, in a patient at the commencement of phthisis in the apex of the lung, we cannot fail to be struck at the successive transition from the soft, easy, mellow character of the inspiratory murmur to the characters of roughness and dryness which it presents as we ascend—a change which becomes still more perceptible according as the tubercular infiltration increases.

2. *Expiratory murmur.*—This, the second part of the respiratory murmur, invariably presents, in the first stage of phthisis, an increase both in its intensity and duration. This increase is constant and regular. The characters of roughness, difficulty, and dryness, are as observable in the expiratory as in the inspiratory murmur. The alterations in the *timbre* of these murmurs, M. Fournet considers to be very important in establishing the diagnosis of the first stage of phthisis. They consist at first of a souffle a little *clearer* than the natural souffle of inspiration or expiration; they then pass on to the *resonant, blowing,* and *bronchial* timbre. The bronchial timbre admits of a *first, second,* and *third degree;* it then becomes *cavernous* or *amphoric,* according to the extent of the cavity. These changes, having arrived at the bronchial character, still belong to the first stage of phthisis; but beyond this, they appertain to the subsequent stages. Even though the cavernous and amphoric characters may be absent, we must not necessarily conclude the non-existence of cavities.

### Prevention of Phthisis.

Mr. Gilbert lays down the following indications:—1st. To hinder the development, in children, of the original predisposition to the disease, by removing from the parents such causes as may be capable of inducing accidental phthisis, or of calling forth the development of hereditary phthisis.

2nd. To prevent the development of the acquired predisposition, by removing from the individual such unhealthy circumstances as may bring on this predisposition to phthisis.

3rd. When once this predisposition to phthisis, whether original or acquired, has become developed, to prevent the process of tuberculization from taking place and localizing itself in the lungs, by combating the disturbances of the function of nutrition, which, we have already shewn, occasion and keep up this process, at the same time that we must remove from the patient such local exciting causes, as may have the effect of determining this morbid movement to the pulmonary organs.

4th. When pulmonary phthisis is established, to prevent, by the adoption of the preceding means, the formation of new tubercles, and so to keep the disease within its first limits of development and severity.

5th. If possible, to make the disease retrograde, by endeavouring to cause the tubercles already formed to disappear, by some other means than that of softening.

To accomplish these very desirable results, he advises judicious matrimonial alliances—attention to the functions of the uterus—attention to diseases of the respiratory system—attention to diseases of the digestive and cutaneous apparatus—fresh air and plenty of light—and a proper choice of habitation and climate.

The last chapter is on the Treatment of Consumption. Mr. Gilbert runs over the various remedies that at different times have been proposed. He speaks highly, and we agree with him, of *Counter-irritants*. He recollects one case of tubercular phthisis, where a young lady was the unfortunate sufferer, and who, as it were to consummate her miseries, was dreadfully scorched on the upper part of her body, through her dress accidentally taking fire. Here, then, was counter-irritation to an extreme; but what was the result? This lady was quickly relieved of her phthisical symptoms, and is now alive and well, never having had any return of her former distressing cough, or other attendants on tubercles in the lungs. She however, to this day, submits to prophylactic treatment. Among other modes of counter-irritation, Mr. Gilbert recommends the common nettle. He is favourable to *emetics*. He is favourable to *inhalation* of volatile substances:—" First, to allay acute inflammation in the lungs and air-passages, in which case a sedative must of course be used. I have found none more useful under such circumstances than the preparations of opium, combined with warm water, and inhaled through an instrument such as Mudge's or Scudamore's Inhaler. The process of inhalation ought to last for five minutes each time, and be repeated every fourth hour, care being taken by the patient not to become exposed to a cold atmosphere after the inhalation. Secondly.—When there exists an obstinate chronic inflammation in the lungs or air-passages, gentle stimulants ought to be inhaled, so as to excite fresh action in the parts. Tar, so strongly recommended by Sir Alexander Crichton, is in such cases not unfrequently extremely beneficial. It may be best administered by heating the tar in a vessel over a spirit lamp, a small proportion of subcarbonate of potass being previously added, to neutralize any pyroligneous acid which the tar may contain. The heat from the lamp ought to be moderate, and the vapour diffused in a chamber to which the patient may frequently repair, and adjoining the room he may generally occupy, care being taken to keep both apartments at an equal temperature. When it can possibly be avoided, the tar ought never to be volatized in the bedroom of the patient, as it adheres to the furniture, and when it becomes offensive to the patient, he cannot avoid the nuisance; in addition to which, by living constantly amongst it, the effect soon becomes lost."

Mr. Gilbert patronises small bleedings. He makes a variety of observations of a judicious description on different remedial measures, diet, and so forth, for which we must refer to the original.

It will be obvious that the chief feature of Mr. Gilbert's work, is the prominence which he gives to a stage anterior to the deposition of tubercles, anterior, in short, to that which is usually called and treated as the first stage of phthisis. And whether disposed or not to accept his theory in all its details, the judicious physiologist and pathologist must allow that, practically as well as speculatively, this is the high and right ground to be taken. It matters little what we term this preparatory state, whether tubercular cachexy, or any thing else, the truth should be present to the minds of all practical men that there is such a stage, and that it is *the* stage for care, and for hopeful, perhaps successful, precautionary measures. If Mr. Gilbert succeeds in directing the attention of the public and the profession to *that*, he will have achieved some good, whatever becomes of his theoretical views.

